# Systematic Reviews and Synthesis without Meta-Analysis on Hydrotherapy for Pain Control in Labor

**DOI:** 10.3390/healthcare12030373

**Published:** 2024-02-01

**Authors:** Elena Mellado-García, Lourdes Díaz-Rodríguez, Jonathan Cortés-Martín, Juan Carlos Sánchez-García, Beatriz Piqueras-Sola, Juan Carlos Higuero Macías, Raquel Rodríguez-Blanque

**Affiliations:** 1 Research Group CTS-1068, Andalusia Research Plan, Junta de Andalucía, 18014 Granada, Spain; emg2684@gmail.com (E.M.-G.); cldiaz@ugr.es (L.D.-R.); jcortesmartin@ugr.es (J.C.-M.); bpiquerassola@gmail.com (B.P.-S.); rarobladoc@ugr.es (R.R.-B.); 2 Costa del Sol Health District, Servicio Andaluz de Salud, Junta de Andalucía, 29640 Fuengirola, Spain; 3 Department of Nursing, Faculty of Health Sciences, University of Granada, 18016 Granada, Spain; 4 Virgen de las Nieves University Hospital, Servicio Andaluz de Salud, Junta de Andalucía, 18014 Granada, Spain; 5 Costa del Sol, Servicio Andaluz de Salud, Junta de Andalucía, 29603 Marbella, Spain; jcarlos.higuero.sspa@juntadeandalucia.es; 6 San Cecilio University Hospital, Servicio Andaluz de Salud, Junta de Andalucía, 18016 Granada, Spain

**Keywords:** water immersion, labor stage, second, evidence-based medicine, pregnant women, delivery, obstetric, analgesia, pain perception

## Abstract

Background: Although there is scientific evidence regarding the use of water immersion during labor, this evidence is primarily focused on the first stage of labor. There is limited scientific evidence on water immersion during the second stage of labor. Objective: The objective of this study was to conduct a comprehensive systematic review and synthesis of contemporary evidence related to water birth, with a specific focus on the second stage of labor. Methods: A systematic review of the scientific literature published between January 2018 and October 2023 was carried out. A synthesis of the results was conducted following the Synthesis without Meta-Analysis (SWiM) guidelines. PubMed, Scopus, and the Cochrane Library were utilized as information sources. The search strategy was designed using the keywords “immersion” and “parturition”, along with their relevant synonyms. Inclusion criteria encompassed studies employing randomized controlled trials (RCTs), systematic reviews, and quantitative and qualitative approaches focusing on pregnant women undergoing water immersion at any stage of the labor process. Results: Eleven articles were selected: two systematic reviews (one quantitative and one qualitative), five cohort studies, one case–control study, one cross-sectional observational study, and two qualitative studies. A thorough assessment of the methodology was performed using several specific tools: the Cochrane RoB 2 (Risk of Bias 2) tool for systematic reviews, JBI Critical Appraisal Checklist for Qualitative Research for qualitative studies, STROBE for observational descriptive studies, and CASPe for qualitative studies. The results provided fundamental insights that will contribute to conceptual standardization regarding the effects of water birth on maternal and fetal health. Additionally, a synthesis of the results was performed concerning types of delivery, analgesia use, pain perception, and maternal satisfaction with the water birth experience. Conclusions: In this study, we conclude that the results regarding delivery types, labor durations, and analgesia use found in the literature, along with statistically significant maternal/fetal effects, are crucial for making recommendations regarding the use of water during labor in any of its stages if the woman desires it safely.

## 1. Introduction

Numerous scientific articles have delved into water birth, seeking to provide answers to associated questions. The benefits of aquatic immersion during the initial stage of labor have been meticulously documented, supported by scientific evidence substantiating the safety of the procedure for both the parturient and the neonate [1]. The use of birthing pools during labor offers several benefits for both the mother and the fetus. Firstly, immersion in warm water provides relief from pain and discomfort by reducing the perception of pain and relaxing the mother’s muscles [2,3], thereby alleviating tension and stress. Additionally, the buoyancy of water facilitates movement and finding comfortable positions, aiding the baby’s rotation and the progression of labor. Fetal flexion is improved, maximizing maternal pelvic diameters and easing the birthing process. The use of birthing pools is associated with a reduced need for epidural analgesia and other pharmacological pain relief, as well as a decrease in medical interventions such as labor induction, episiotomy, and cesarean section [4]. Many women report an increased sense of control and satisfaction during childbirth when using these pools. Furthermore, there is a lower risk of severe perineal tears and better postpartum recovery due to the relaxation provided by warm water. Regarding the fetus, no increased risks have been observed, with low rates of neonatal infection and normal Apgar scores for babies born in the water [2,4]. It is crucial to note that these benefits primarily apply to uncomplicated deliveries, and the use of birthing pools should be carried out under the appropriate supervision and care of trained healthcare professionals. Every woman and pregnancy is unique, so it is essential to discuss birthing options with the healthcare provider to make informed decisions [2]. However, it is pertinent to note that certain studies, by focusing on the first stage of labor, do not address the expulsive phase, limiting the extent and robustness of available evidence [5].

In a recent study conducted by Burns et al. [6], the conclusion was reached that there are no adverse associations in outcomes for either the mother or neonate in relation to water birth. However, the use of water immersion during labor stages does not always have unanimous support from obstetrics and gynecology institutions. While widespread acceptance of this practice prevails among professionals attending low-risk pregnancies in the United Kingdom [7], the American College of Obstetricians and Gynecologists (ACOG) in the United States focuses on the inadequacy of randomized clinical trials [8]. It is relevant to note that such research has inherent limitations, such as obtaining samples conditioned by the willingness of participating women, limited scientific production specifically on the second stage of labor, and the absence of blinding in trials [9]. Most published studies take the form of observational studies, which—by concluding in favor of benefits and questioning the harm of water birth—diverge from some current guidelines. At the same time, they propose the possibility for hospitals to implement safe water birth programs for low-risk women, in accordance with the wishes of the parturients [9].

Research has suggested a wide range of potential benefits associated with the aquatic environment. Firstly, it is relevant to highlight that the properties of water allow buoyancy and freedom of movement. Rest and activity in this medium can lead to a reduction in fear, anxiety, and pain perception during the birthing process. Working in water has been observed to optimize childbirth physiology by releasing endogenous endorphins and oxytocin [6]. Water exposure also involves coping with contractions, as the water temperature promotes maternal relaxation, concurrently reducing stress-related hormones and pain perception, resulting in lower demand for analgesia by the woman [5,10].

In the Spanish context, the current clinical practice guideline on normal birth care prescribes hot water immersion as an effective method for relieving pain during the late stage of the first stage of labor [11]. The compelling need to undertake this systematic review arises from the limited availability of information regarding water immersion during the second stage of labor. In response to a recent assessment highlighting the scarcity of specific scientific evidence in this context, the lack of conclusive data on the effects of water immersion during the expulsion phase becomes evident. The report underscores the absence of a national protocol addressing this practice specifically, emphasizing the importance of conducting a comprehensive review to better understand the associated benefits and risks. The systematic review stands as a crucial initiative to address existing gaps in the scientific literature, providing a more robust foundation for future clinical and policy decisions in this particular aspect of the childbirth process [12,13].

In light of the above, the following research question is raised: What is the current scientific evidence regarding the benefits and risks associated with water births, particularly during the second stage of the birthing process? To address the research question, the purpose of our study was to conduct a comprehensive systematic review and synthesis of contemporary evidence related to water birth, with a specific focus on the second stage of labor. Our intention is to contribute significantly to the scientific body by critically examining the benefits and risks inherent in water immersion during childbirth, as outlined in the selected scientific articles for review.

## 2. Materials and Methods

The preparation of this report followed the methodology of a systematic review using the guidelines provided by the Preferred Reporting Items for Systematic Reviews and Meta-Analyses (PRISMA) [14] guidelines and the Synthesis Without Meta-Analysis (SWiM) extension [15]. The review focused on scientific literature published from January 2018 to October 2023. The present systematic review was conducted according to a specific protocol, which is available on the website: http://www.crd.york.ac.uk, accessed on 10 March 2023, under the registration number CRD42023399625.

### 2.1. Eligibility Criteria

We included articles utilizing systematic review methodologies, along with quantitative and qualitative studies concentrating on pregnant women undergoing water immersion at any phase of the childbirth process. No language restrictions were applied during the selection process.

### 2.2. Information Sources

The bibliographic inquiry was conducted in the Scopus, PubMed, and Cochrane Library databases. The structuring of the search language was based on Medical Subject Headings (MeSH) terms and Health Science Descriptors (DeCS). The descriptors used were “immersion” and “parturition”, employing the Boolean operators AND and OR to refine and expand the search, respectively.

### 2.3. Search Strategy

In Table 1, the search strategy employed for the execution of this research in each of the consulted databases is displayed. The precise date on which this search was conducted is included, thus providing a detailed description of the methodology used in the information-gathering process.

### 2.4. Data Extraction Process

Following the execution of the search strategy, the identified articles were transferred to the Mendeley web platform using the Mendeley Web Importer tool. Subsequently, the documents were organized into folders and classified according to the original database, followed by the removal of duplicates.

The studies considered for inclusion in this analysis encompassed systematic reviews as well as quantitative and qualitative studies, with the aim of evaluating the effects of water birth on maternal–fetal health published between January 2018 and October 2023. Two reviewers (E.M.-G. and J.C.-M.) independently conducted the review of the title, abstract, and keywords of each study identified in the search, applying inclusion and exclusion criteria. For those potentially eligible studies, the same procedure was applied to the full-text articles. Discrepancies between reviewers were resolved through discussion or if necessary by a third reviewer (J.C.S.-G.).

The collection of data related to quality, patient characteristics, interventions, and relevant outcomes was independently carried out by two different reviewers (R.R.-B. and B.P.-S.).

### 2.5. Data Collection Process and Collected Data

Two evaluators, identified by their initials, conducted data extraction for each included article, addressing aspects such as authors, article type, participants, objectives, interventions, measurement instruments, key findings, and conclusions. The selection of studies used in the results was independently performed by two researchers (E.M.-G. and J.C.-M.) through the review of titles and abstracts. In this process, the decision was made to select studies that—in addition to meeting the inclusion criteria—provided recent and conclusive evidence regarding water birth and its maternal–fetal outcomes.

Additionally, an evaluation of the strengths and weaknesses inherent to each study was carried out focusing on the primary outcome, i.e., the use of water during childbirth compared to women who experienced conventional delivery, expressed, for example, in terms of the type of delivery (percentage).

In the Results section, the article selection process is explained in detail. Studies that did not meet the inclusion criteria or presented inconclusive evidence were excluded from the results.

### 2.6. Risk of Bias in Individual Studies

To conduct the methodological assessment of the articles included in this study, a comprehensive analysis of the design, methodology, and study type of each work was undertaken to select the most appropriate methodological evaluation scale for each case.

The risk of bias in the systematic review was assessed using the RoB 2 (Risk of Bias 2) tool [16].

For the qualitative systematic review, the JBI Critical Appraisal Checklist for Qualitative Research [17] was employed, recommended for meta-aggregative reviews. Developed by the Joanna Briggs Institute for critical and interpretive research, this checklist consists of 10 questions with response options: “yes”, “no”, “unclear”, or “not applicable”.

Descriptive observational studies underwent methodological quality assessment using the STROBE checklist [18]. This statement, consisting of 22 points, ensures that studies meet all necessary elements for quality in a descriptive article. The 22 items are organized into seven classic sections (IMRD: Introduction, Methods, Results, Discussion), with two preceding sections (title and abstract) and one following (funding). For qualitative studies, the CASPe critical appraisal tool [19] was utilized, enabling the assessment of the quality of a qualitative study in three respects: rigor, credibility, and relevance. It comprises 10 questions with three possible responses: “yes”, “no”, or “unclear”.

### 2.7. Results Synthesis

The methodological characteristics of each study (such as study designs, intervention types, or outcomes) were too diverse to produce a meaningful summary estimate of effect, so the SWiM guidelines were used for the synthesis of the selected articles [15].

Considering the information gathered in this review, results emerge that provide a set of fundamental premises. These premises are outlined as key elements to standardize concepts related to the effects of water birth on both maternal and fetal health. Furthermore, a synthesis of the results is achieved, encompassing aspects such as types of delivery, analgesia use, duration of labor, maternal outcomes, neonatal outcomes and pain perception associated with the water birth experience.

## 3. Results

The collected information provides essential premises to standardize concepts regarding the impacts of water birth on maternal and fetal health. The synthesis covers key aspects, such as types of delivery, analgesia use, pain perception, and maternal satisfaction, consolidating the understanding of results derived from heterogeneous studies.

Throughout the review, a total of 11 studies employing various research methodologies were identified. These comprised two systematic reviews (one quantitative and one qualitative in nature), five cohort studies, one case–control study, one cross-sectional observational study, and two qualitative investigations. The inclusion of this variety of methodologies allowed for a comprehensive approach to different variables related to water birth.

The obtained results exhibited variability among the studies, reflecting notable differences in the presented evidence. This variability is attributed to the diversity in the methodological approaches employed, as well as the specific objectives pursued by each study.

**Figure 1:**
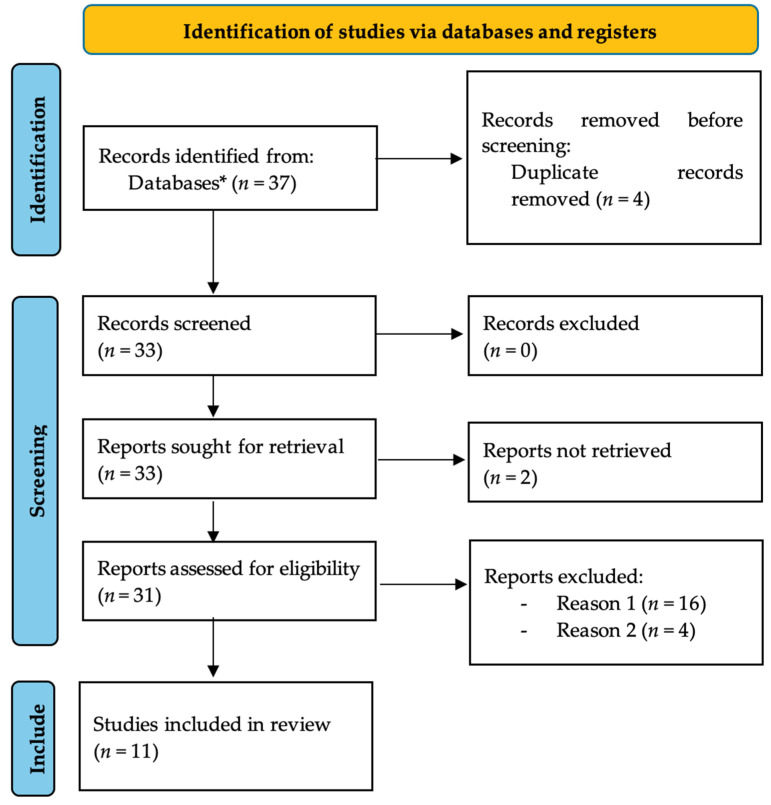
Flow diagram. * Out of the 37 articles, 9 were from PubMed, 26 from Scopus, and 4 from the Cochrane Library.

Figure 1 depicts the flowchart of the article selection process in this systematic review.

The risk-of-bias assessment of the articles yielded the following results. The review by Cluett et al. (2018) [5] generally appears to have a low risk of bias. The primary sources of bias arise from the scarcity of data (limited statistical power) rather than from methodological issues or difficulties in presenting results. The outcomes of the methodological quality analysis following the STROBE checklist were not lower than 13 (Czech et al. (2018) [20]), while the remaining studies exhibited a low risk of bias, scoring between 17 and 20 points. Regarding studies employing qualitative methodology [21,22], both displayed a low risk of bias.

Table 2 shows the summary of the results according to the SWiM guidelines.

Table 3 summarizes various studies related to water birth or immersion during labor. The main objectives addressed were to assess the effects of water on the delivery method, pain levels, oxidative stress in newborns, women’s experiences, and maternal/neonatal outcomes. The results show that water can reduce the need for analgesia, decrease pain, and increase relaxation and the perception of control during childbirth. It does not seem to affect delivery methods or increase risks for mothers or babies. Lower oxidative stress was reported in newborns from the water group. Women’s experiences were mostly positive, highlighting both physical and emotional benefits. Some expressed fears or complications, mainly associated with infrastructure. On the other hand, many received limited information about the water option. The examined studies showed good maternal and neonatal outcomes after water birth or immersion, with no differences compared to standard care and even some advantages, such as less pain and greater satisfaction. In general, the summarized research concludes that water birth or immersion appears to be a safe and effective alternative to enhance the experience for many women. However, there is a need to strengthen information processes and care protocols.

## 4. Discussion

This review has been conducted to investigate the effects of water immersion at various stages of the childbirth process, focusing on maternal and fetal/neonatal outcomes, as well as identifying its influence on the mode of delivery. The practice of water birth has been the subject of study for over four decades in multiple countries, enabling the compilation of significant results within the scope of this review.

Throughout the review, a total of 11 studies employing various research methodologies were identified. These comprised two systematic reviews (one quantitative and one qualitative in nature), five cohort studies, one case–control study, one cross-sectional observational study, and two qualitative investigations. The inclusion of this variety of methodologies allowed for a comprehensive approach to different aspects related to water birth.

The obtained results exhibited variability among the studies, reflecting notable differences in the presented evidence. This variability is attributed to the diversity in the employed methodological approaches, as well as the specific objectives pursued by each study. The assessment of results emerges as a complex process in which the nature of the research and its specific goals influence the interpretation of the effects of water immersion in various dimensions of childbirth.

### 4.1. Types of Delivery

Three specific studies [27,28] in the present analysis addressed the incidence of different types of delivery, observing no statistically significant differences between the groups that underwent water birth and those who opted for conventional methods. This conclusion aligns with the findings of the systematic review by Cluett et al. [5], which examined a total of 15 trials, of which 8 were considered and evaluated. Across these 8 trials, covering a total population of 3663 subjects, no substantial differences were evidenced between the groups regarding various modes of delivery, whether spontaneous vaginal, instrumental, or cesarean.

This pattern of results suggests consistency in the reviewed literature, indicating a lack of significant differences in outcomes related to types of delivery between those who underwent water immersion during the childbirth process and those who did not. The alignment of these results with the systematic review by Cluett et al. [5] strengthens the coherence and robustness of the evidence gathered on this specific theme.

### 4.2. Use of Analgesia

During the first stage of labor, the systematic review conducted by Cluett et al. [5] reveals a discrepancy in the use of epidural analgesia between women who opted for water immersion and those who did not. According to this study, in the group of women who experienced water birth, a lower proportion received epidural analgesia compared to the groups that did not use water. However, no significant differences were identified in the use of epidural analgesia or the use of pethidine/narcotics between the groups.

On the other hand, Barry et al. [27] present contrasting findings regarding the administration of analgesia during the first stage of labor. According to this study, women who participated in the water immersion group showed a lower likelihood of using epidural analgesia compared to those in the control group who did not use water immersion. The observed difference was substantial, with a significantly less epidural analgesia use in the water immersion group (15.9%) compared to the control group (48.9%).

This discrepancy between studies highlights the variability in results and suggests that the relationship between water immersion during the first stage of labor and the use of epidural analgesia may depend on multiple factors, including specific care protocols and characteristics of the studied population. Addressing these differences is necessary to fully understand the influence of water immersion on decisions related to analgesia during the first stage of labor.

### 4.3. Duration of Labor

The present review of studies, which assessed the duration of the childbirth process through specific investigations [22,24,28], has concluded that there are no statistically significant differences in the overall duration of labor between the groups that used water immersion and those that did not. This consistency in results suggests that water immersion may not have a substantial impact on the total duration of the childbirth process according to the parameters evaluated in the selected studies.

However, it is pertinent to note that there are studies highlighting the potential positive influence of water immersion on the duration of labor through its relaxing effects. In particular, studies like that of Cluett et al. [5] and other research have underscored the relaxing nature of water and its potential to reduce the duration of labor.

In contrast, a prospective study evaluating myeloperoxidase levels during water immersion [24] did not identify statistically significant differences in the total duration of labor. However, it was observed that the average duration of the first stage of labor was shorter in the water immersion group. This observation was supported by other studies, such as those by Neiman et al. [21] and Ulfsdottir et al. [26], who reported that women in the water birth group experienced a shorter duration of the first and second stages of labor.

The interpretation of these results suggests that the possible presence of favorable conditions during water birth that could counteract stress and tension, potentially facilitating the progress of labor. This approach could be considered a significant aspect to explore in future research on the effects of water immersion during the childbirth process [31].

### 4.4. Maternal Outcomes

The analyzed studies, particularly those included in the systematic review by Cluett et al. [5], have consistently concluded that there is insufficient evidence to establish significant differences in third and fourth-degree tears associated with water birth. This finding highlights the lack of conclusive evidence regarding the influence of water immersion on the incidence of higher-grade tears during the childbirth process.

Regarding specific research on tears [25], some detailed studies have not found statistically significant differences between the groups that used water immersion and those that did not [27,28]. This lack of difference in tear incidence suggests that the practice of water birth does not appear to be associated with a significant increase in vulnerability to this type of injury.

As for postpartum hemorrhage, the results of the reviewed studies also point to the absence of significant differences between the groups that opted for water immersion and those that did not. Specific investigations [26,27] have not found statistically significant differences in postpartum blood loss between the two groups. Similarly, the systematic review by Cluett et al. [5] concludes that there is insufficient evidence to determine the effect of water immersion on postpartum blood loss.

These findings indicate the need for a more detailed and rigorous evaluation of the available evidence, as well as the importance of future research to address more specifically and conclusively the potential effects of water immersion on outcomes such as tears and postpartum hemorrhage during the childbirth process.

### 4.5. Neonatal Outcomes

In the analysis of neonatal events recorded in the reviewed studies, the results of our systematic review indicate that no statistically significant differences were found between the groups of women who used water immersion during childbirth and those who opted for conventional methods. The studies referenced as [5,27,28] support this conclusion by not observing significant disparities in the evaluated neonatal events.

Eckert et al. [22] notes a higher use of resuscitation in the group of neonates born to women who used water immersion, although it emphasizes that increased neonatal infectious morbidity has not been associated with bath use. Additionally, Apgar scores show no significant differences between the groups. On the other hand, the study by Uzunlar et al. [28] reveals that epidural analgesia impaired oxidative stress status and reduced neonatal Apgar scores, resulting in a higher admission rate to the Neonatal Intensive Care Unit (NICU) for neonates whose mothers used epidural analgesia. This study, comparing three groups (the water-use group, the non-water-use and no analgesia group, and the epidural analgesia-use group), identified statistically significant differences in NICU admissions for neonates whose mothers used epidural analgesia during childbirth.

According to Ulfsdottir et al. [26], no differences were observed in Apgar scores between the groups, and none of the neonates in the conventional childbirth group were transferred to the NICU, while none of the neonates in the water birth group required NICU transfer.

Cluett et al. [5] reports on perinatal death, admission to the neonatal intensive care unit, and neonatal infection, concluding that the reviewed studies do not show clear differences between the groups in these outcomes.

Overall, these results suggest that in general, water immersion during childbirth does not seem to be associated with significant differences in neonatal events, although some studies highlight specific aspects that may require more detailed consideration in future research.

### 4.6. Pain and Childbirth Experience

Immersion in warm water during childbirth has been associated with a relaxing effect that facilitates neurohormonal interactions, relieves pain, and optimizes the progression of labor [32]. This effect is attributed to the reduction of sensory stimulation, decreasing the likelihood of stress hormone secretion [25], revealing that women who used hydrotherapy during labor experienced pain relief, avoiding the need for analgesia. It suggests that water immersion can be an effective nonpharmacological method for pain relief during childbirth [25,32].

Regarding the relationship between water birth and pain reduction, some results from our review do not support the statistical association between water birth or water immersion and a significant decrease in pain severity [20]. In a randomized controlled trial conducted by Eckert et al. [22], measurements of mean scores for the experienced pain impression and appropriateness of pain relief at 24 and 48 h, as well as 8 months after childbirth, showed no significant differences between water birth and conventional childbirth groups.

Pain relief has been qualitatively explored as one of the physical benefits of water immersion during childbirth. According to surveys, many women described water as soothing for the vulva and perineum during the second stage of labor, highlighting improvement in the pain experience and its decrease between contractions.

Pain management is linked to childbirth experiences, and qualitative studies have addressed various topics related to water birth [23,29,30]. Qualitative evidence suggests that women giving birth in water perceive having more control and empowerment by not using pharmacological analgesia, thus achieving positive childbirth experiences [24]. Other qualitative studies, such as Dado et al. [29], positively describe the water birth experience, strongly linking it to women’s ability to follow their instincts. Additionally, research like Carlsson et al. [30] concludes that women giving birth in water experience physical and psychological benefits, but highlights the need for better equipment and sufficient information both prenatally and during childbirth. Authors like Reviriego-Rodrigo et al. [23] emphasize the perspective of midwives, concluding that water births must be safely ensured, with adequate resources and training for midwives, along with the implementation of standardized protocols to allow all pregnant women to safely choose water use during childbirth with satisfactory results.

### 4.7. Implications for Clinical Practice

According to the analyzed documentation, the following recommendations are proposed for the clinical practice of healthcare professionals.

- Provide comprehensive information about the potential benefits and risks of water birth to all pregnant women.
- Equip birthing facilities with suitable facilities and equipment to conduct safe water births (birthing tubs, water heaters, etc.).
- Establish protocols that enable healthcare personnel to determine low-risk criteria for opting for this alternative (maternal and fetal health status, gestational week, fetal position, etc.).
- Develop consensus birth plans between the medical team and the pregnant woman addressing potential scenarios and when to resort to other options.
- Standardize the entry into the birthing pool as closely as possible to the onset of active labor to maximize its analgesic benefits, considering the possibility of delivering outside the water in cases of complications or exhaustion during labor.
- Establish means of continuous support and accompaniment by trained personnel throughout immersion and childbirth.

## 5. Conclusions

The growing demand among women to undergo water birth highlights the importance of ensuring optimal human resources and infrastructure to guarantee the safety of both the mother and the baby, supported by scientific evidence. Findings from a literature review suggest that such variables as the type of delivery, duration of labor, and analgesia usage are crucial for making recommendations on the safe use of water during childbirth. While water immersion may offer benefits, it is emphasized that secure implementation necessitates appropriate conditions, resources, and training, underscoring the significance of providing women with the option of this practice.

## Figures and Tables

**Table 1:** Search Strategy.

Source	Search String	Limits	Search Date
SCOPUS	(TITLE-ABS-KEY (immersion OR “Immersions” OR “Submersion” OR “Submersions”) AND TITLE-ABS-KEY (parturition OR “Parturitions” OR “Birth” OR “Births” OR “Childbirth” OR “Childbirths”) AND TITLE-ABS-KEY (pregnant AND women))	-	1 to 15 November 2023
PUBMED	((immersion[MeSH Terms]) OR (“Immersions” OR “Submersion” OR “Submersions”) AND ((y_5[Filter]) AND (humans[Filter]) AND (female[Filter]) AND (alladult[Filter] OR youngadult[Filter] OR adult[Filter] OR middleagedaged[Filter])))	Humans Female Adult	5 to 25 November 2023
COCHRANE LIBRARY	#1: MeSH descriptor: [Immersion] explode all trees #2: MeSH descriptor: [Parturition] explode all trees #1 AND #2 Filter: Clinical Trials	Clinical trials	20 November 2023

**Table 2:** Summary of results according to the SWiM guidelines.

Article	Benefits	Risks
Cluett et al. (2018) [5]	1. Decrease in the use of analgesia during the first stage of labor (dilating period). 2. Shorter duration of the first stage of labor. 3. Better level of maternal satisfaction in the expulsive period in water.	1. Isolated cases of sepsis (a severe infection) have been reported in the newborn, and also maternal cases due to *Pseudomonas aeruginosa*, difficulty in thermoregulation, hypovolemic shock, hyponatremia, respiratory difficulty and hypoxic–ischemic encephalopathy, resulting in some lethal cases. Given the limitations of many studies, it is difficult to establish the incidence of complications.
Reviriego-Rodrigo et al. (2023) [23]	1. Pain relief. 2. Feeling of control. 3. Feeling of relaxation. 4. Improved mobility. 5. Improved satisfaction.	1. Concerns about the baby’s safety. 2. Concerns about water hygiene. 3. Concerns about the ability of professionals to detect and manage complications.
Ibanoglu et al. (2022) [24]	1. Decreased pain. 2. Reduction in the need for epidural analgesia.	No significant risks were reported.
Camargo et al. (2018) [25]	No significant benefits were reported.	No significant risks were reported.
Ulfsdottir et al. (2019) [26]	1. Pain relief. 2. Feeling of control. 3. Feeling of relaxation. 4. Improved mobility. 5. Improved satisfaction.	No significant risks were reported.
Barry et al. (2020) [27]	1. Less need for epidural analgesia. 2. Greater maternal satisfaction.	No significant risks were reported.
Czech et al. (2018) [20]	No significant benefits were reported.	No significant risks were reported.
Neiman et al. (2020) [21]	1. Less need for epidural analgesia. 2. Shorter duration of labor. 3. Lower episiotomy rate.	No significant risks were reported.
Uzunlar et al. (2021) [28]	No significant benefits were reported.	No significant risks were reported.
Dado et al. (2022) [29]	1. Pain relief. 2. Feeling of control. 3. Feeling of relaxation. 4. Improved mobility. 5. Improved satisfaction.	No significant risks were reported.
Carlsson et al. (2020) [30]	1. Pain reduction. 2. Greater maternal satisfaction.	1. Concerns about the baby’s safety. 2. Concerns about water hygiene. 3. Concerns about the ability of professionals to detect and manage complications.

**Table 3:** Selected articles for the systematic review [5,23,24,25,26,27,28,29].

Authors	Type	Objectives	Outcomes	Punch line
Cluett et al. (2018) [5]	Systematic Review.	Assessing the effects of water immersion during labor and/or delivery (first, second, and third stages of labor) on women and their infants.	Comparing water immersion at any stage of labor, no clear differences were found in type of delivery, blood loss, or neonatal complications. Fewer women in the immersion group received an epidural, with no differences in ICU admission, neonatal infections, type of delivery, or mortality. Maternal satisfaction was higher in the water immersion group during the second stage of labor.	Labor in water may reduce the need for an epidural. This review found no evidence that water birth increases the risk of adverse outcomes for women or their newborns.
Dado et al. (2022) [29]	Qualitative study	Its primary aim is to comprehend realities through personal experiences, feelings, and individuals’ perspectives.	The women in this study described it positively and it was strongly associated with women’s perception of having the ability to trust their instincts, facilitated by the soothing effect of the water. All women in the study described the birth of their babies as a positive birth experience. Few women were informed about the option of using the pool during the prenatal period.	Improving the implementation of waterbirth as a care option for women in Ireland. Contributes to increased maternal and family satisfaction, and improves the quality of care and overall birth experience for women.
Carlsson et al. (2020) [30]	Qualitative study	Explore retrospective accounts of benefits, negative experiences, and preparatory information related to water births.	Physical benefits were highlighted as facilitating labor progression, buoyancy and pain relief; psychological benefits as greater relaxation and control in a nonmedicalized and safe environment. Negative experiences were identified as (a) equipment-related problems due to tub construction and problems associated with water immersion, and (b) fears and concerns related to water birth. Lack of general and specific information about water births was reflected.	The lack of adequate equipment in Swedish maternity units underlines the need to question the current routines and resources in Swedish maternity units to better adapt them to the needs of pregnant women.
Uzunlar et al. (2021) [28]	Prospective Cohort Study	Investigate the cord blood level of copeptin, total serum oxidant (TOS), antioxidant (TAS), interleukin (IL)-1, IL-6, and oxytocin levels following labor with water immersion, epidural analgesia, and vaginal delivery without pain relief.	There were no statistically significant differences between the three groups for duration of the first and second stages of labor, total duration of labor, labor intervention rate, the presence of perineal trauma and lactation status. APGAR scores at 1 and 5 min were significantly lower in group 2 compared to groups 1 and 3. TAS, TOS and copeptin levels were significantly higher in the epidural group than in the control and water groups. The need for admission to the neonatal intensive care unit (NICU) was significantly higher in the epidural group (*p* = 0.011), with rates of 3.3%, 20%, and 2.3% in groups 1, 2, and 3, respectively.	Epidural analgesia is associated with elevated levels of oxidants and antioxidants, as well as less satisfactory neonatal outcomes compared to conventional water birth.
Reviriego-Rodrigo et al. (2023) [23]	Systematic review and thematic synthesis of qualitative evidence were conducted.	Investigate the experiences of women and midwives with water immersion during labor.	The reasons for choosing waterbirth are prior knowledge of positive experiences, recommendations, seeking relaxation and anxiety reduction, feeling of comfort and well-being, desire for natural childbirth and pain relief. The advantages of waterbirth include a lower likelihood of perineal tearing, a shorter active phase of labor, no increased risk of neonatal mortality compared to conventional delivery, no adverse effect on the newborn’s general condition (Apgar score) and no increased risk of infection for the newborn.	The findings underscore the feasibility and efficacy of water immersion as a safe option during childbirth, and highlight the importance of adequate resources and rigorous protocols, backed by a culture of support for this practice by midwives.
Ibanoglu et al. [24]	Case–control study.	Compare the levels of myeloperoxidase (MPO) in umbilical cord blood samples from mothers undergoing water immersion versus conventional labor.	The mean duration of the first stage of labor was shorter in the water immersion group, as was the visual analogue scale (VAS) pain score of 7 vs. 9. Myeloperoxidase (MPO) values were significantly lower in the water immersion group than in the control group (*p* = 0.004).	The findings of this study demonstrate that labor pain can be effectively reduced through water immersion during the first stage of labor. Regarded as an analgesic method, it is a convenient and comfortable approach that does not entail complications associated with anesthesia and does not require the involvement of an anesthesiologist.
Camargo et al. (2018) [25]	Cross-sectional and observational quantitative study of women in water immersion, noncomparative.	Analyze the maternal and neonatal outcomes of 90 low-risk pregnant women who gave birth in the water at São Bernardo Hospital.	Apgar scores were greater than 7, 93.7% of the women showered for nonpharmacologic pain relief, and 94.3% had no desire to leave the pool. Only 1.1% requested pharmacologic measures for pain relief. There was a decrease in cervical dilatation time and a shorter duration of the expulsion phase. Regarding neonatal outcomes, 97% maintained a normal fetal heart rate (between 110 and 160 beats per minute) during maternal immersion.	Water birth was satisfactory and safe for the women/couples and newborns. There were no negative effects on neonatal outcomes. On maternal outcomes, immersion influenced the duration of labor and was a crucial element in pain relief due to its relaxing effects and the freedom of movement and positions it allowed.
Ulfsdottir et al. (2019) [26]	Prospective cohort study	Compare the childbirth experiences between women who had a water birth and those who had a conventional, uncomplicated delivery.	Women who had water births scored significantly higher in the “Self-capacity” domain and lower in the “Professional support” domain. They reported less pain and higher control scores during the second stage of labor. These women felt less dependent on the midwife.	Overall, waterbirth appears to empower women, enhancing their experience and possibly reducing their need for midwifery assistance.
Barry et al. (2020) [27]	Prospective cohort study	Examine childbirth outcomes for women and babies after water immersion solely for labor or for both labor and delivery.	Water immersion during childbirth was associated with more spontaneous vaginal deliveries and less use of epidurals, but also with a slight increase in the risk of postpartum hemorrhage. Women who chose water immersion more frequently experienced babies with higher birth weight, but there were no significant differences in adverse neonatal outcomes. Additionally, initiation and exclusivity of breastfeeding were higher in this group.	Water immersion appears to be a safe alternative for low-risk women and is rated very positively by women in terms of birth experience.
Czech et al. (2018) [20]	Prospective cohort study	Assess the effectiveness of both pharmacological and nonpharmacological pain relief methods and compare their outcomes.	There were no statistically significant differences in childbirth pain levels between women who attended parent education classes and those who did not. Perineal massage did not reduce the frequency of perineal incisions, and episiotomy did not impact pain intensity in the study participants. Among those who underwent episiotomy, the majority were nulliparous. No significant pain level differences were noted between epidural and gas analgesia groups in the first stage of labor, but epidural analgesia effectively reduced pain during the second and third stages. Water immersion yielded the highest satisfaction levels.	Water birth did not show a statistically significant reduction in pain intensity, but it was well-received and associated with the highest satisfaction among women. Water immersion remains the most accepted non-pharmacological pain relief option, unlike TENS, which was associated with the lowest satisfaction level in the study.
Neiman et al. (2020) [21]	Prospective cohort study	Generate evidence regarding maternal and neonatal outcomes associated with water immersion during labor and delivery.	Water birth did not show significant risks for newborns, and mothers who chose water birth reported high satisfaction. However, a higher incidence of postpartum hemorrhage was observed in this group, despite a reduction in the duration of the early stages of labor. These findings emphasize the importance of weighing the benefits and risks of water birth, as well as the necessity for proper training for perinatal care professionals in various settings.	This study adds to the existing evidence on the risks and benefits of water birth for women and newborns, emphasizing the importance of proper training for perinatal care providers handling births in various settings.

## Data Availability

Data are available on request from the corresponding author.
